# Daily antibiotic cost of nosocomial infections in a Turkish university hospital

**DOI:** 10.1186/1471-2334-5-5

**Published:** 2005-01-31

**Authors:** Dilara Inan, Rabin Saba, Filiz Gunseren, Gozde Ongut, Ozge Turhan, Ata Nevzat Yalcin, Latife Mamikoglu

**Affiliations:** 1 Department of Infectious Diseases and Clinical Microbiology, Faculty of Medicine, Akdeniz University, Antalya, Turkey; 2 Department of Medical Microbiology, Faculty of Medicine, Akdeniz University, Antalya, Turkey

## Abstract

**Background:**

Many studies associated nosocomial infections with increased hospital costs due to extra days in hospital, staff time, extra investigations and drug treatment. The cost of antibiotic treatment for these infections represents a significant part of hospital expenditure. This prospective observational study was designed to determine the daily antibiotic cost of nosocomial infections per infected adult patient in Akdeniz University Hospital.

**Methods:**

All adult patients admitted to the ICUs between January 1, 2000, and June 30, 2003 who had only one nosocomial infection during their stay were included in the study. Infection sites and pathogens, antimicrobial treatment of patient and it's cost were recorded. Daily antibiotic costs were calculated per infected patient.

**Results:**

Among the 8460 study patients, 817 (16.6%) developed 1407 episodes of nosocomial infection. Two hundred thirty three (2.7%) presented with only one nosocomial infection. Mean daily antibiotic cost was $89.64. Daily antibiotic cost was $99.02 for pneumonia, $94.32 for bloodstream infection, $94.31 for surgical site infection, $52.37 for urinary tract infection, and $162.35 for the other infections per patient. The treatment of *Pseudomonas aeruginosa *infections was the most expensive infection treated. Piperacillin-tazobactam and amikacin were the most prescribed antibiotics, and meropenem was the most expensive drug for treatment of the nosocomial infections in the ICU.

**Conclusions:**

Daily antibiotic cost of nosocomial infections is an important part of extra costs that should be reduced providing rational antibiotic usage in hospitals.

## Background

Nosocomial infections are frequent complications of hospitalization and also an important public health problem in developing countries, as well as in developed ones. The socioeconomic impact, ie, prolongation of hospitalization, mortality, and cost of these infections adversely effects patients and nations' economic well-being [1]. The cost of nosocomial infections includes increased length of hospital stay, staff time, laboratory cultures of pathogens and antimicrobial treatment [2-4]. Although, cost of antimicrobial treatment is an important part of health expenditure, data on this subject are extremely limited in Turkey.

The aim of our study was to determine daily antibiotic cost of nosocomial infection per infected patient in a university hospital.

## Methods

### The hospital setting

Akdeniz University Hospital is a 600-bed tertiary referral centre in Antalya, Turkey, treating 27 000 patients per year. The study was conducted in six adult medical and surgical intensive care units (ICUs) with a total of 51 ICU beds. Neonatal ICU was not included in the study. Since 1993, the institutional policies of hospital infection control have been implemented by infection control team.

### Definitions and study population

In our hospital, routine prospective, active surveillance of nosocomial infections in all ICUs is performed by one infection control nurse, supervised by an infection control physician. Nosocomial infections are defined using the Centers for Disease Control and Prevention criteria [5,6]. We do not follow patients for signs of infection after discharge unless they are readmitted to the hospital.

Between January 1, 2000 and June 30, 2003, all inpatients hospitalized in one of the adult ICUs were included in this study. Data on antimicrobial treatment were recorded for patients aged 15 or above presenting with only one nosocomial infection. For all patients included in the study, the following were recorded: age, sex, infection site, microbiologic data, antimicrobial therapy and antibiotic cost.

### Measurement of costs

In our ICUs, all antimicrobial prescriptions are recommended by an infectious disease consultant. Antimicrobial agents prescribed only for therapeutic indications were recorded. The daily antibiotic cost was calculated in US dollars based on June 2003 prices of antimicrobial agents provided by the hospital pharmacy. The daily antibiotic cost per infected patient was calculated by the multiplication of box price and number of daily doses that was used for that infection. Two costs were calculated for each antimicrobial; the minimal cost (min) was based on the lowest recommended parenteral daily dose and the maximal cost (max) was based on the highest recommended parenteral daily dose.

## Results

Between January 1, 2000, and June 30, 2003, a total of 8460 patients were admitted to the adult ICUs. Overall, 817 patients developed 1407 episodes of nosocomial infections, accounting for an infection rate of 16,6%. Among them, 233 patients (mean age:50,1; sex ratio female:male 0,49) had only one nosocomial infection. Mean daily antibiotic cost was found $89,64 per infected patient ($8,56 to $359,28).

Among the sites of nosocomial infections, urinary tract infections had the lowest daily antibiotic cost per infected patient (Table 1). The mean daily antibiotic cost for pneumonia was the highest of all sites, but patients with bloodstream infection reached the highest range of daily cost ($31,31 to $359,28). In addition, mean daily antibiotic cost was found $162,35 for seventeen other infections including postoperative meningitis, mediastinitis, and empyema.

**Table 1:** Daily antibiotic costs according to the sites of nosocomial infections.

Site of nosocomial infections	Number of patients	Range of daily cost per infected (US$)	Mean (US$)
Pneumonia	111	10,02–250,74	99,02
Bloodstream infections	28	31,31–359,28	94,32
Surgical site infections	11	17,12–204,74	94,31
Urinary tract infections	66	8,56–228,68	52,37

Out of 233 patients, 206 patients had microbiologically documented infections, 177 patients were infected by a single pathogen while 29 patients were diagnosed as having polymicrobial infections (Table 2). *Pseudomonas aeruginosa *was the most prevalent bacteria followed by *Klebsiella *spp. and *Acinetobacter *spp.. *P. aeruginosa *infections had the highest overall daily antibiotic cost per infected patient than other pathogens. Among 21 *Staphylococcus aureus *strains 15 (72%) were resistant to methicillin. The overall daily antibiotic cost of methicillin resistant *S. aureus *(MRSA) infections was two to three times higher than infections with susceptible strains. However, median daily cost per infected patient for MRSA infections were lower than susceptible strain infections.

**Table 2:** Daily antibiotic cost according to the pathogens.

Pathogens	No.	Overall daily cost (US$)	Range of daily cost per pathogen (US$)	Median (US$)
*Pseudomonas aeruginosa*	60	5567,06	17,98–204,74	100,04
*Acinetobacter *spp	36	4951,06	17,98–359,28	92,47
*Stenotrophomonas maltophilia*	4	310,99	31,31–100,04	89,82
*Klebsiella *spp	37	3104,22	10,02–179,64	79,6
*Enterobacter *spp	10	961,79	60,42–139,08	77,28
*Escherichia coli*	23	1652,57	10,02–179,64	60,41
*Proteus *spp	3	49,04	13,24–49,04	49,04

*Staphylococcus aureus*				

Methicillin-susceptible (MS)	6	476,22	10,02–142,2	74,52
Methicillin-resistance (MR)	15	1188,1	35,88–142,2	71,1

CoNS*				

MS-CoNS	1	49,64	16,34–49,64	49,64
MR-CoNS	3	148,92	17,52–49,64	49,64
*Enterococcus *spp	16	1141,25	32,29–142,2	49,64
*Candida *spp	21	235,52	8,56–38,64	8,56
No pathogen identified	27	3166,75	10,02–359,28	114,6

* Coagulase negative *Staphylococcus*

Among the 350 antibiotic prescriptions for nosocomial infections, piperacillin-tazobactam and amikacin were the most prescribed antibiotics (Table 3). Carbapenems especially meropenem were the most expensive drugs.

**Table 3:** Daily cost of antimicrobial agents for nosocomial infections.

Antimicrobial agents	Number of prescriptions	Daily cost per infected (US$)	
		
		Min	Max
Betalactams			

Ampicillin-sulbactam	20	23,92	71,76
Piperacillin-tazobactam	48	85,95	114,6
Ticarcillin-clavulanate	4	14,9	22,35

Carbapenems			

Imipenem	31	100,04	150,06
Meropenem	23	179,64	359,28

Cephalosporins			

Cefepime	26	38,64	77,28
Ceftazidime	18	35,82	71,64
Cefoperazone-sulbactam	16	40,28	80,56
Cefazoline	10	10,02	20,04
Ceftriaxone	3	19,7	39,4

Aminoglycosides*			

Amikacin	52	-	7,96
Netilmicin	21	-	24,48
Tobramycin	3	-	18,56

Fluoroquinolones			

Ciprofloxacin	18	49,04	98,08
Ofloxacin	2	25,74	51,48
Levofloxacin	1	41,07	82,14

Glycopeptides			

Vancomycin	13	49,64	87,27
Teicoplanin	12	71,1	142,2

Other antibiotics			

Clarithromycin	4	11,57	23,14
Trimethoprim-sulphamethoxazole	2	4,48	8,96
Metronidazole	2	5,51	10,04

Antifungal agents			

Fluconazole	21	8,56	34,24
Total	350	8,56	359,28

* dosage given as once-daily.

## Disscussion

Cost is an important factor which determines the physician's choice of medication to treat patients in spesific stiuations. In this study, we tried to demonstrate the daily cost of antimicrobial treatment of nosocomial infections according to site of infection, pathogen and antimicrobial agent.

In different studies, economical analysis regarding costs attributable to nosocomial infections has been evaluated and reported between $1018 to 2280 per infected patient [7-9]. Jarvis et al reported that the estimated average costs of nosocomial infections were $558 to 593 for each urinary tract infection, $2734 for each surgical site infection, $3061 to 40000 for each bloodstream infection, and $4947 for each pneumonia [1].

Daily cost of antimicrobial treatment has been reported to be a significant extra cost attributable to nosocomial infections. In this study, we found an average daily antibiotic cost of $89,64 per nosocomial infection. It is clear that cost of overall antibiotic treatment for a period of approximately 10–15 days is $900 to $1350. Prolongation of hospital stay has been the major extra cost attributable to nosocomial infections in many reports [2-4], but in comparative case-control study from our country, Yalcin et al. [8] found that cost of antibiotic therapy of $1190 per infected patient, accounted for about 75% of the total extra cost. This finding may be due to the high prices of antibiotics in Turkey. To calculate the true costs of antibiotic therapy, hidden costs arising from intravenous administration, labor, serum antibiotic assay, monitoring hematological and biochemical indices and adverse effects of antibiotics must be considered [10]. The present study does not include these relevant "hidden costs" that could substantially modify the total cost of an antibiotic treatment. Although, hidden costs were not calculated, an average daily antibiotic cost of a single nosocomial infection is found to be markedly high in our hospital.

This result is within the limits reported by other large economic studies, suggesting that our data is comparable to those found in other countries and with other assessment methods. In a French prevalence survey, Astagneau et al.[11] reported an average daily antibiotic cost between FF 520 to 1085 (about $86 to $160) per nosocomial infection. French et al.[12] and Haley et al.[13] reported an average cost of antibiotic treatment of $190 and between $72 to $128 per nosocomial infection, respectively. In Turkey, Yalcin et al.[14] found that daily antibiotic cost of nosocomial infections was $70 per patient.

The daily antibiotic cost varies markedly according to site of infection. Our study has demonstrated that pneumonia and bloodstream infections were associated with the highest daily antibiotic costs as reported in other studies [11,13,14]. Surgical site infections had also high daily antibiotic cost in our study. In their case-control study, Coello et al. reported that antibiotic therapy for surgical patients was the second most significant contributor to cost [15]. In the present study, nosocomially infected patients that had only one nosocomial infection were considered for analysis. Clearly, antimicrobial treatment of patients with multiple nosocomial infections might be much more expensive.

*P. aeruginosa *infections had the highest daily antibiotic cost followed by other non-fermentative bacilli. Infections caused by *P. aeruginosa *are difficult to treat because of its virulence and relatively limited choice of effective antimicrobial agents, so, these infections often require combination therapy. Emergence of resistance in *P. aeruginosa *has been associated with increased morbidity, mortality, and costs [16]. On the other hand, although the overall antibiotic cost of MRSA infections was higher than infections with susceptible strains, the daily antibiotic cost per infected patient with MRSA was lower with susceptible strain infections. MRSA infections are treated by glycopeptides which cost less than beta-lactams in our country. Astagneau et al reported that the daily antibiotic cost of multi-resistant bacterial infections such as multi-resistant *P. aeruginosa *infections, was 20% higher than susceptible infections, but the daily antibiotic cost per infected patient for MRSA infections was not higher than for susceptible strain infections [11].

Expensive antibiotics, such as piperacillin-tazobactam, carbapenems, cefepime, ciprofloxacin, teicoplanin were prescribed more commonly than the cheaper agents such as ampicillin-sulbactam, ceftriaxone or ofloxacin in our ICUs. These expensive antibiotics were mainly prescribed for resistant and severe gram-negative nosocomial infections, such as ventilator-associated pneumonia and postneurosurgical meningitis in ICU. Physicians may be forced to choose empirical antibiotic therapy with broad spectrum antimicrobials by increasing bacterial multi-resistance.

In conclusion, mean daily antibiotic cost was found $89,64 per nosocomial infection in our ICUs and nosocomial pneumonia had the highest daily antibiotic cost per infected patient. It is clear that cost of antibiotic therapy of nosocomial infections is an important part of extra cost attributable to nosocomial infection. Approximately one third of nosocomial infections are preventable by full implementation of the current infection control guideline recommendations [17]. Each institution should develope empirical antibiotic guidelines according to its own local nosocomial infections data. Infection control measures, such as education of health care workers regarding antimicrobial agents and resistance; isolation of patients infected with multi-resistant organisms, should be implemented to reduce infections and expensive antibiotic prescriptions.

## Competing interests

The author(s) declare that they have no competing interests.

## Authors' conributions

DI collated and analyzed the data, participated in the study design and was principal writer of manuscript. ANY conceived the study. GO carried out the laboratory studies. RS, FG, OT and LM participated in the patient management. All authors read and approved the final manuscript.

## Pre-publication history

The pre-publication history for this paper can be accessed here:


